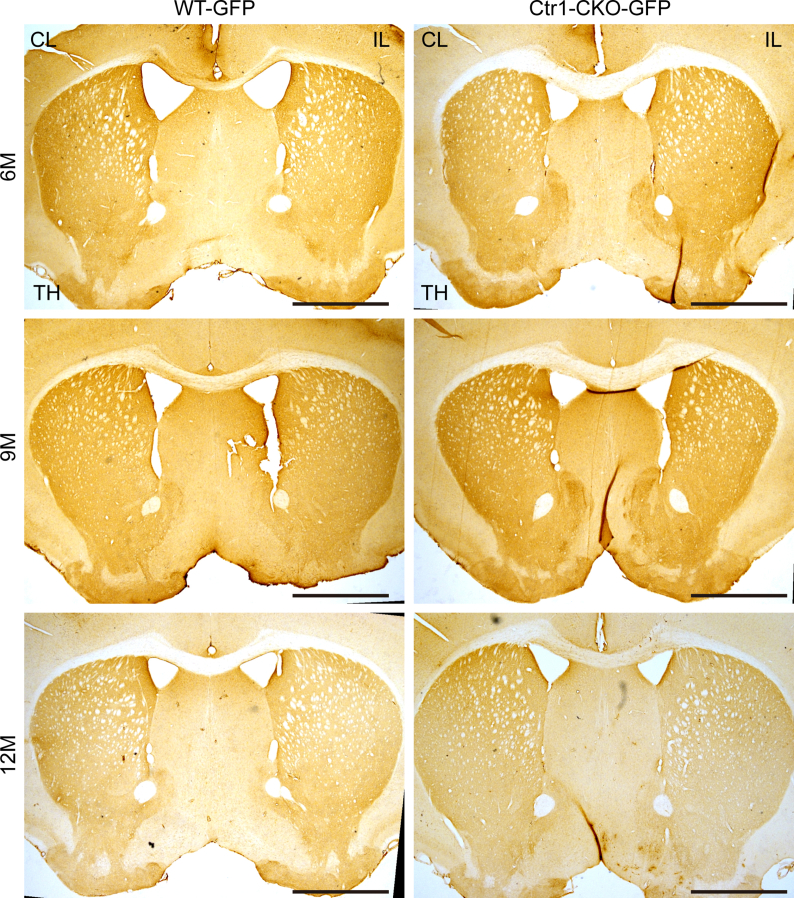# Inhibition of copper transporter 1 prevents α-synuclein pathology and alleviates nigrostriatal degeneration in AAV-based mouse model of Parkinson's disease

**DOI:** 10.1016/j.redox.2020.101795

**Published:** 2020-11-12

**Authors:** De-Hai Gou, Ting-Ting Huang, Wen Li, Xin-Di Gao, Caroline Haikal, Xin-He Wang, Dong-Yan Song, Xin Liang, Lin Zhu, Yong Tang, Chen Ding, Jia-Yi Li

**Affiliations:** a Institute of Neuroscience, College of Life and Health Sciences, Northeastern University, Shenyang, 110169, China; b Institute of Health Sciences, China Medical University, Shenyang, 110122, China; c Neural Plasticity and Repair Unit, Wallenberg Neuroscience Center, Department of Experimental Medical Science, Lund University, BMC A10, 22184, Lund, Sweden; d Department of Histology and Embryology, Chongqing Medical University, Chongqing, 400016, China; e Laboratory of Stem Cells and Tissue Engineering, Chongqing Medical University, Chongqing, 400016, China

**Keywords:** Copper transporter 1, α-Synuclein, Neurodegeneration, Nigrostriatal system, Parkinson's disease

## Abstract

The formation of α-synuclein aggregates is a major pathological hallmark of Parkinson's disease. Copper promotes α-synuclein aggregation and toxicity *in vitro*. The level of copper and copper transporter 1, which is the only known high-affinity copper importer in the brain, decreases in the substantia nigra of Parkinson's disease patients. However, the relationship between copper, copper transporter 1 and α-synuclein pathology remains elusive. Here, we aim to decipher the molecular mechanisms of copper and copper transporter 1 underlying Parkinson's disease pathology. We employed yeast and mammalian cell models expressing human α-synuclein, where exogenous copper accelerated intracellular α-synuclein inclusions and silencing copper transporter 1 reduced α-synuclein aggregates *in vitro*, suggesting that copper transporter 1 might inhibit α-synuclein pathology. To study our hypothesis *in vivo*, we generated a new transgenic mouse model with copper transporter 1 conditional knocked-out specifically in dopaminergic neuron. Meanwhile, we unilaterally injected adeno-associated viral human-α-synuclein into the substantia nigra of these mice. Importantly, we found that copper transporter 1 deficiency significantly reduced S129-phosphorylation of α-synuclein, prevented dopaminergic neuronal loss, and alleviated motor dysfunction caused by α-synuclein overexpression *in vivo*. Overall, our data indicated that inhibition of copper transporter 1 alleviated α-synuclein mediated pathologies and provided a novel therapeutic strategy for Parkinson's disease and other synucleinopathies.

## Highlights

• Ctr1 deficiency reduces α-synuclein aggregates *in vitro.*
• Ctr1 deficiency inhibits the level of pathological α-synuclein *in vivo.*
• Ctr1 deficiency prevents nigrostriatal neurodegeneration *in vivo.*
• Ctr1 deficiency alleviates motor dysfunction caused by α-synuclein *in vivo.*

## Graphical abstract

**Figure:**
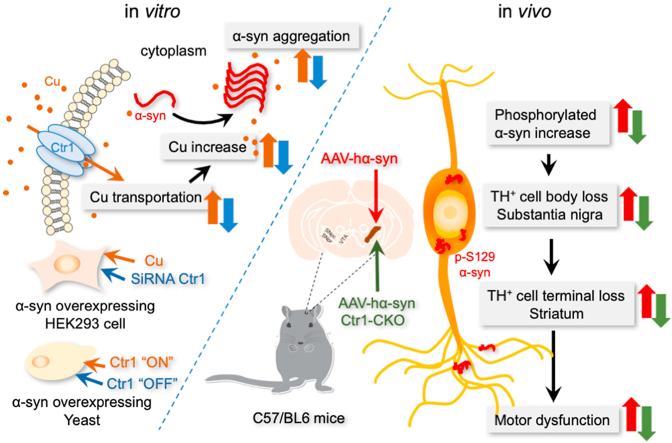

## Abbreviations

AAS: atomic absorption spectroscopy
AAV: adeno-associated virus
α-Syn: α-synuclein
CCS: Cu chaperone for superoxide dismutase
CL: contralateral
Ctr: copper transporter
Ctr1: copper transporter 1
Ctr1-CKO: Ctr1 conditional knock-out
Cu: copper
GAL1: galactose-inducible 1
hα-syn: human α-syn
IHC: immunohistochemistry
IF: immunofluorescence
IL: ipsilateral
LBs: Lewy bodies
LNs: Lewy neurites
NC: negative control
PD: Parkinson's disease
pS129-α-syn: phosphorylation of serine129-α-syn
RT: room temperature
SC: synthetic complete
SDD-AGE: Semi-denaturing detergent agarose gel electrophoresis
SN: substantia nigra
SNpc: substantia nigra pars compacta
SNpr: substantia nigra pars reticulata
SOD1: copper/zinc-dependent superoxide dismutase
TH: tyrosine hydroxylase
WB: Western blot
WT: wild type

## 1 Introduction

Parkinson's disease (PD) is the second most common neurodegenerative disease in humans. Loss of dopaminergic neurons in the substantia nigra pars compacta (SNpc) and the presence of Lewy bodies (LBs) and Lewy neurites (LNs) are the two major neuropathological hallmarks of PD. LBs and LNs are primarily composed of α-synuclein (α-syn) [[1], [2], [3]], which is particularly abundant in presynaptic terminals [4,5], where it regulates exocytosis, endocytosis, and synaptic maintenance [[6], [7], [8], [9]]. Many animal studies have shown that pathologic α-syn plays a causative role in the progress of neurodegeneration [[10], [11], [12]], such as loss of dopaminergic neurons and reduction of dopamine in the nigrostriatal system, which ultimately leads to the onset of motor symptoms, e.g. muscle rigidity, resting tremor, bradykinesia and gait impairment [12,13]. Phosphorylation of serine 129-α-syn (pS129-α-syn) is the majority pathologic modification of α-syn and it was considered as a predominant LBs/LNs phenotype, which appears to involve neurotoxicity, aggregation and subcellular distribution [11,[14], [15], [16]]. Additionally, pathologic α-syn propagates in a “prion-like” manner via neural pathways [[17], [18], [19], [20]], which causes more extensive pathological spreading of α-syn. Thus, the molecular mechanism underlying what triggers or facilitates α-syn pathology has been under intense investigations.

Previous studies have extensively elucidated that several metal ions can interact with α-syn to promote aggregation [[21], [22], [23], [24]]. Cu is one of the most relevant metal ion accelerating the aggregation of α-syn, which primarily binds to the N-terminal domain of α-syn *in vitro* [23,[25], [26], [27], [28], [29], [30]]. Cu is also essential for many biological processes in the central nervous system, including neurotransmitter synthesis and release, melanin production, and synaptic signaling [31,32]. Conversely, Cu dyshomeostasis is associated with several neurodegenerative disorders, such as PD, Menkes disease, Wilson's disease, Alzheimer's disease (AD), and amyotrophic lateral sclerosis (ALS) [[33], [34], [35], [36], [37]]. More recently, the clinical investigations have concluded that brain samples from patients with PD showed reduction of Cu level in the substantia nigra (SN) [38,39]. Moreover, the level of Copper transporter 1 (Ctr1) significantly reduced in the SN of PD patients [40], which implied Ctr1 is associated with PD. As we known, Ctr1 is a homo-trimeric plasma membrane protein, which is the only known high affinity and specificity mammalian copper importer [41,42]. Evolutionarily conserved from fungi to humans, Ctr1 is responsible for transporting Cu into the cytoplasm [[43], [44], [45], [46]]. After import, Cu is transported to the downstream functional proteins by specific copper chaperone proteins [35,47,48]. For instance, copper chaperone for superoxide dismutase (CCS) delivers copper to cytosolic copper/zinc-dependent superoxide dismutase (SOD1) [49]. Furthermore, in response to elevated copper, the levels of Ctr1 reduce and Ctr1 endocytosis leads to degradation in mammalian cells [50,51]. In mammals, the gene encoding Ctr1 is essential for embryonic development, heart function, and intestinal Cu absorption [43,44,50], whereas the function of Ctr1 during the development of neurodegenerative disease remain unclear.

In the present study, we deciphered the role of Ctr1 in α-syn mediated pathologies in PD. We first addressed the correlation between Ctr1 and α-syn *in vitro*. Utilizing mammalian cell and yeast strains expressing human α-syn (hα-syn), we demonstrated that increasing Ctr1 levels resulted in an accumulation of intracellular Cu content, which triggers α-syn aggregation, while silencing Ctr1 significantly dampened this process. In the dopaminergic cell-specific Ctr1 conditional knock-out (Ctr1-CKO) mice, we observed that Ctr1 deficiency reduced the level of S129-phosphorylated α-syn, as well as prevented dopaminergic neuronal loss in response to unilateral injection of adeno-associated virus (AAV)-hα-syn in the SN. Moreover, Ctr1 deficiency consistently alleviated the motor dysfunction induced by α-syn dependent pathology. These data showed that Ctr1 deficiency inhibited α-syn-mediated pathologies, suggesting that Ctr1 is a key modulator to α-syn neurotoxicity and it may merit clinical consideration of modifying therapies in PD and other synucleinopathies.

## 2 Materials and methods

### 2.1 Animals

To generate the Ctr1-CKO mice, we used Cre-loxP recombination technology. The Ctr1^flox/flox^ mice [43] (referred to as WT mice) possessing the loxP-flanked *CTR1* gene were bred to TH-Cre mice [52] expressing Cre recombinase under the control of an exogenous tyrosine hydroxylase (TH) promoter, and the offspring (TH-Cre; Ctr1^flox/+^ mice) were crossed with Ctr1^flox/flox^ mice, and TH-Cre; Ctr1^flox/flox^ mice (referred to as Ctr1-CKO mice) were obtained. The mouse lines containing the Ctr1^flox/flox^ transgene were identified by PCR with the primers (forward primer-P1 *5′-AAT GTC CTG GTG CGT CTG AAA-3′* and reverse primer-P2 *5′-GCA GTA GAT AAA AGC CAA GGC-3′*) [43] and genotyping of TH-Cre was carried out by standard approaches using PCR with the primers (forward primer *5′-AAA TGT TGC TGG ATA GTT TTT ACT GC-3′* and reverse primer *5′-GGA AGG TGT CCA ATT TAC TGA CCG TA-3′*) [52]. To detect specific tissue excision of Ctr1 by TH-Cre, P1, P2 and P3 (*5′-AAA AAC CAC TAT TCA GAG ACT G-3′*) were performed [43]. The Ctr1^flox/flox^ mice on C57BL/6J genetic background were a kind gift of Drs Dennis J. Thiele (Duke University School of Medicine, USA) and Yan-Fang Wang (Institute of Animal Sciences of Chinese Academy of Agricultural Science). The TH-Cre mice (C57BL/6J background) were obtained from Jackson Laboratories (JAX stock #008601).

All animal experiments and surgical procedures were conducted in strict accordance with the international guidelines and were approved by the Research Ethics Committee of the College of Life and Health Sciences of Northeastern University (China). All mice were bred with food and water provided *ad libitum* and were housed under a standard 12-h light/dark cycle (on 7 a.m., off 7 p.m.) at room temperature. Sufficient procedures were employed to minimize animal pain or discomfort during the experiments.

### 2.2 Stereotactic injection of the AAV viruses

The vector for production of AAV serotype 5 inducing the overexpression of either human wild-type α-syn or enhanced green fluorescent protein (GFP), driven by the synapsin-1 promoter and enhanced using a woodchuck hepatitis virus posttranscriptional regulatory element (WPRE), were performed as described previously and acquired by Lund University (Sweden) [53]. The genome copy titers for AAV-GFP and AAV- hα-syn were 9.2E14 and 6.0 E14 genome copies/mL, respectively. The viral stock solutions were diluted to the equivalent concentration (3.6E13 genome copies/mL) with 0.9% stroke-physiological saline solution before injection.

Animals were injected with AAV-GFP or AAV- hα-syn vector. The number of different groups was provided in the Supplementary Table 1 (total number was 80 mice). Briefly, all surgical procedures were performed under general anesthesia using 50 mg/kg sodium pentobarbital injected intraperitoneal. Mice were placed in a stereotaxic frame (RWD) and performed a small craniotomy using a 1 mm gauge drill bit at the following coordinates for the right SN: antero-posterior: − 3.10; medio-lateral: − 1.20; dorsa-ventral: − 4.0 relative to the bregma and dural surface according to the stereotaxic atlas [54,55]. Then, 2 μL of either AAV-GFP or AAV-hα-syn solutions were injected using a 10 μL Hamilton microsyringe at a rate of 0.2 μL/min and the needle was left in place for an additional 5 min before it was slowly retracted. Mice were sutured and returned to normal housing conditions. As a result, the number of injection viral particles is 7.2E10. The mice were assessed for motor function relative behavioral tests after inoculation of 8 weeks.

### 2.3 Behavioral tests

*Open Field Test*. A small open field (40 × 40 × 40 cm) was used to evaluate spontaneous motor activity and exploratory behaviors. Briefly, mice were carried to the test room for 30 min to adapt to the new experimental surrounding. Then, the mice were placed into the center arena and permitted to freely explore for 10 min, while being recorded using an overhead digital camera (SONY) connected to the automated video-tracking system (Harvard Apparatus, model: SMART® v 3.0). The apparatus was wiped with 70% ethyl alcohol and allowed to dry between tests.

*Wire Hang Test*. The wire hang test was conventionally used to determine general motor function and deficits in rodent models [17]. The mice were placed on a mesh wire (30 × 30 cm), which was then inverted and suspended 30 cm above the cage. The latency before falling was recorded. A trial was complete when the mice fell or 10 min had passed. The test was performed on 3 days with three trials per session.

### 2.4 Tissue collection

After the behavioral tests, all mice were deeply anaesthetized with sodium pentobarbital and perfused with 0.9% heparinized saline. For western blot experiments, the tissues need to be quickly frozen in liquid nitrogen and stored at -80 °C. For immunohistochemical staining experiments, the tissues were dissected and fixed using ice-cold 4% paraformaldehyde (PFA) in 0.1 M PBS overnight and then stored in cryoprotectant (30% sucrose containing 20% ethylene glycol and 0.05% sodium azide in 0.1 M PBS) at 4 °C.

### 2.5 Immunostaining procedure

The brains were cut to six series at 30 μm-thick coronal sections using sliding microtomes (Leica SM2010 R) and stored in cryoprotectant (30% sucrose containing 20% ethylene glycol and 0.05% sodium azide) in a -20 °C freezer. Immunostaining was performed as free-floating method.

For immunohistochemistry (IHC), the sections were immersed in antigen retrieval buffer (0.04% citric acid and 0.3% trisodium citrate dihydrate, pH 6.0) for 1 h at 60 °C. Endogenous peroxidase was blocked by incubation with 3% hydrogen peroxidase and 10% methanol in 0.1 M PBS for 30 min at room temperature. Next, the sections were incubated with blocking solution (10% normal serum with 1% BSA and 0.3% Triton-X100 in 0.1 M PBS) for 1 h at room temperature, then incubated with primary antibodies (Supplementary Table 2) overnight at 4 °C. Then, sections were incubated in the paired biotinylated secondary antibodies (Supplementary Table 2) at room temperature for 2 h. For IHC imaging, the common avidin-biotin-peroxidase complex was applied; the sections were incubated in ABC solution (Vector Lab, Cat.# VEPK-6100) for 1 h at room temperature and were visualized with 0.03% diaminobenzidine in 0.05 M tris-HCl (pH 7.5) for 3 min. Additionally, the sections were rinsed with 0.1 M PBS for 3 × 10 min at every interval. The sections were mounted and dehydrated sequentially 5 min with 70, 80, 90, 95, 100% ethanol and dimethyl benzene for 10 min twice, then mounted and coverslipped with neutral balsam.

For immunofluorescence (IF), after the antigen retrieval process as IHC, the sections were incubated by blocking solution as IHC at room temperature for 1 h, then incubated with primary antibodies (Supplementary Table 2) overnight at 4 °C. Then, the sections were incubated the matched fluorescence second antibodies (Supplementary Table 2) at room temperature for 1 h. The sections were stained with DAPI for 5 min at room temperature, then mounted with antifade solution and coverslipped for confocal microscopic analyses (Leica TCS SP8). In addition, the sections were rinsed with 0.1 M PBS for 3 × 10 min at every interval.

### 2.6 Cell cultures and immunofluorescence

We used the HEK293 cells stably expressing hα-syn labeled with DsRed (HEK293-hα-syn-DsRed), which was generated as previously described [54]. The cells were maintained in DMEM (Gibco) supplemented with 10% FBS (BI) and 1% penicillin/streptomycin (P/S) at 37 °C and 5% CO_2_. CuSO_4_ was applied to the fresh medium with 50 or 100 μM.

Cells were cultured on the cover glass (Fisher brand) for IF. In brief, samples were fixed with 4% PFA (room temperature) for 15 min, permeabilized with 0.25% Triton X-100 for 15 min, and blocked with 3% BSA for 1 h at room temperature. Cells were incubated with primary antibodies (Supplementary Table 2) overnight at 4 °C. The following day, the samples were stained with fluorophore-conjugated secondary antibodies (Supplementary Table 2) for 2 h at room temperature and DAPI for 5 min before mounted with antifade solution. Samples were rinsed three times with 0.1 M PBS between each incubation period.

### 2.7 RNA interference

In this experiment, we used siRNA targeting Ctr1 (siRNA-Ctr1, #s3377 and #s3379, Thermo Fisher Scientific) and negative control siRNA (Thermo Fisher Scientific) to transfect the HEK293-hα-syn-DsRed cell line. The cells were seeded at a density of 30% and incubated for 24 h in 6-well plates. Transfections were performed with 50 nM siRNAs per well using Lipofectamine® RNAiMAX (Invitrogen). After 48 h, the efficiency of transfection was evaluated by Western blot analysis. The procedure of diluting the siRNA followed standard protocols provided by Thermo Fisher Scientific.

### 2.8 Yeast strains

*S. cerevisiae* BY4741 was cultured in synthetic complete (SC) broth or SC agar medium using standard procedures. The overlapping sequences of hα-syn-GFP were introduced into p426GPD as *HindIII*-*SalI*-digested products of PCR amplification. The *S. cerevisiae CTR1* gene was cloned into pESC as *BamhI*-*ApaI*-digested products. Yeast strains carrying hα-syn-GFP were maintained in SC medium lacking uracil. Transformations of yeast were cultivated with lithium acetate. Yeast strains carrying the galactose-inducible Ctr1 constructs were pre-grown in SC medium with 1% raffinose and induced with 2% galactose.

### 2.9 Western blot analysis

Cell samples were rinsed three times with sterile PBS and were scraped into radioimmunoprecipitation assay lysis buffer (Beyotime) containing 2 mM PMSF (Beyotime) and 1% protease inhibitor cocktail (Sigma-Aldrich) incubated for 30 min at 4 °C. The samples were centrifuged at 13,000 rpm for 10 min at 4 °C to obtain the supernatants. Mice tissues frozen and stored in a -80 °C refrigerator were fully immersed in the radioimmunoprecipitation assay lysis buffer containing 2 mM PMSF and 1% protease inhibitor cocktail, homogenated by ultrasonic crashing, and centrifuged at 15,000 rpm for 30 min at 4 °C. Then, the protein concentration was measured by BCA kit (Beyotime). The samples were prepared with 5X SDS loading buffer and boiled 5 min at 95 °C. 20 μg of total protein were loaded per line. Samples were separated by 4–20% SDS-PAGE for 75 min at 40 mA. Gels were transferred onto polyvinylidene difluoride membranes (Millipore) during 90 min at 60 V. After blocking in 5% skimmed milk (Millipore) in Tris-saline 0.05% Tween-20 (TBST), the membranes were hybridized with the primary antibodies (Supplementary Table 2) overnight at 4 °C. Corresponding secondary HRP antibodies (ECL Rabbit IgG, #NA934; ECL Mouse IgG, #NA931; GE Life Sciences) were diluted in TBST 5% milk. Detection was enhanced by ECL kits (#180–5001, Tanon) and revealed with chemiluminescence imaging analysis system (Tanon 5500). Bands were analyzed by ImageJ software and normalized to the corresponding GAPDH or β-actin signal and compared to the matched control group.

### 2.10 Semi-denaturing detergent agarose gel electrophoresis

Semi-denaturing detergent agarose gel electrophoresis (SDD-AGE) is a common method to detect prion and prion-like polymers and amyloid conformational variants [56,57]. Cell samples were harvested by centrifugation at 800 rpm for 5 min at room temperature and resuspended in lysis buffer (100 mM Tris, pH 7.5; 50 mM NaCl; 4 mM PMSF; 2% protease inhibitor cocktail) and vortexed on high speed for 2 min. The supernatant was recovered by centrifugation at 13,000 rpm for 15 min at 4 °C. Protein concentration was determined using BCA protein assay. The samples were enriched with 4X sample buffer (2X TAE, 20% glycerol, 8% SDS, bromophenol blue to preference) and incubated for 5 min at room temperature, while one sample was boiled 5 min at 95 °C as the negative control. 200 μg of total protein were loaded per well in the gel (1.5% agarose in 1X TAE solution containing 0.1% SDS) and ran 15–18 h at 20 V at 4 °C. The transferring procedure was the same as TurboBlotter System (GE Healthcare) that was used in a previous experiment [57]. It should be noted that, after transfer overnight, the membrane could be processed by standard Western blot analysis.

### 2.11 Copper analysis

Fresh mouse brain tissues were digested with 500 μL nitric acid (Sigma) at 100 °C for 30 min. After the samples returned to room temperature, they were diluted 20 times with 0.1% nitric acid (Sigma). Then, the samples were measured using AAS (ZEEnit700P, Analytikjena, Germany) as previously described [58].

### 2.12 Stereological cell counting and optical densitometry analysis

For quantification of the nigrostriatal pathway with staining for α-syn, pS129-α-syn and TH, each marker antibody was stained with a series of 30 μm-thick coronal sections in six series. Every sixth section covering the entire extent of the SN or striatum was included in the counting procedure, as described previously [53]. The IHC images were performed with Lionheart FX 180404A (version 3.05.11, BioTek) on Lionheart™ FX automated microscope (color bright field, 4X objective, BioTek), NIS-Elements BR imaging system (software version 4.12, Nikon) on Nikon microscope (Nikon Eclipse 80i, 2X, 10×, 20× objective) and Leica DM4000B light microscope (5X, 10×, 20× objective). The IF staining was visualized by Leica application suite (LAS) X on confocal microscopy (Leica TCS SP8).

Assessment of the number of TH-positive neurons in the SN was made according to the optical fractionator principle [[59], [60], [61]], using the stereological system including a computer-assisted mapping and cell quantification program (Stereo Investigator, MBF Bioscience) coupled to an Imager M2 microscope (ZEISS). A coefficient of error of <0.10 was accepted [62]. A full series of sections (3 sections) per animal was used to count. The interesting counting regions were drawn at 5X magnification. Quantifications were performed at 100X (oil objective), using the settings of counting frame (X = 60 μm, Y = 60 μm) and grid size (X = 134 μm, Y = 134 μm) and guard zone height (1 μm) and dissector height (9 μm). Then, the number of TH-positive neurons was calculated according to the following equation:

$$N=\sum Q^{-}\times \frac{1}{SSF}\times \frac{1}{ASF}\times \frac{1}{HSF}$$

where *N* is the total number of cells, and ∑*Q*^−^ is the actual counting cell number in the specimens, and *SSF* is the section sampling fraction, and *ASF* is the area sampling fraction, and *HSF* is the thickness sampling fraction. In the present experiment, *SSF* was 1/6, and *ASF* is 20%, and *HSF* was calculated as dissector height/mean thickness. Finally, data were represented as a percentage of the respective contralateral side.

Quantification of α-Syn positive signal in the SNpc was measured by optical densitometry, five images were performed with Leica DM4000B light microscope (20X objective) in every SNpc slide per mice. Eight images were captured for quantification of TH-positive fiber and α-Syn positive signal in the striatum. Using ImageJ, the images were converted to an 8-bit format and corrected for non-specific background staining by subtracting values obtained from the cortex to obtain the suitable intensity threshold and measured the intensity value. Data were presented as a normalized values of the intensity corresponding area from the contralateral side. pS129-α-syn of the SNpc were counted in the whole SNpc slides of one series on Leica DM4000B light microscope (10X objective). The fluorescence images were reconstructed by the maximum projection from Z-stack pictures. In addition, for each experiment, images in each channel were captured using the same acquisition. All analyses were done blind to different groups. The number of animals used in each experimental group was indicated in the figures.

In the experiment of HEK293-hα-syn-DsRed supplied with CuSO_4_, specimen analyses were performed with Leica application suite (LAS) X on confocal microscopy (Leica TCS SP8). We analyzed the percentage of α-syn accumulated cells in randomly selected five hundred α-syn positive cells for each condition. The data were shown as the fold change normalized by control group. In the yeast model, specimen analyses were performed with NIS-Elements imaging system (software version 4.12, Nikon) on conventional epifluorescence microscope (Nikon Eclipse 80×i, 100× oil objective). The data were represented as the fold change of the percentage of α-syn accumulated cells in three hundred α-syn positive cells (each group) randomly selected.

### 2.13 Statistical analysis

Cell experiments were carried out using three independent biological duplications with three technical replicates. In the animal studies, it was ensured at least three mice in each group. All data were mainly analyzed using ImageJ (National Institutes of Health) and GraphPad Prism 8.0. Two-way analysis of variance (ANOVA) and one-way ANOVA were employed. Tukey's multiple comparisons test followed two-way ANOVA and one-way ANOVA. All values are presented as the mean ± standard error of the mean. p values *p < 0.05; **p < 0.01, ***p < 0.001 were considered to be significant.

## 3 Results

### 3.1 Copper exacerbates α-syn inclusions in mammalian cells

Cu interacts with cellular α-syn through the high affinity domains located in the N-terminus of α-syn and forms a complex to participate in the mechanisms of α-syn aggregation [23,27,29,30,[63], [64], [65]]. To confirm this effect *in vitro*, we utilized HEK293 cells stably expressing DsRed-tagged hα-syn [54]. After treatments with CuSO_4_ at different concentrations (0, 50 and 100 μM), we first examined the intracellular Cu levels by atomic absorption spectroscopy (AAS) and the level of CCS. As we known, CCS is a key copper chaperone protein in cytoplasm, which delivers copper to cytosolic SOD1. Furthermore, the CCS level is negatively correlated with Cu concentration in the cytoplasm [35,43]. When the intracellular Cu concentration increased (Supplementary Fig. 1A), the CCS level declined (Supplementary Fig. 1B and C), indicating the internalization of extracellular Cu. In high Cu level cells (50, 100 μM), we detected 3–4-fold elevation of the percentage of cells containing α-syn inclusions, compared to cells under Cu-depletion conditions (Fig. 1A and B). In addition, the immunoblot of α-syn exhibited the gradual enhancement of accumulated α-syn in a Cu dose-dependent manner (0–100 μM) (Supplementary Fig. 1D), which indicated that Cu facilitated the formation of α-syn aggregates. Next, we further study the aggregated α-syn under high Cu level. After 48 h treated with 50 μM CuSO_4_, the monomer α-syn level was decreased (Fig. 1C, shorter exposure time, and Fig. 1F), whereas the level of oligomer α-syn obviously increased 4-fold than from control group (Fig. 1C, longer exposure time image, and Fig. 1G). Whether did Cu trigger α-syn to form amyloid-like structure? Interestingly, the level of amyloid-like α-syn was remarkably increased at high levels of Cu (50 μM) detected by SDD-AGE (Fig. 1D and H). Together, our data demonstrate that Cu triggers the aggregation of α-syn, possibly leading to α-syn mediated pathology.

**Fig. 1:**
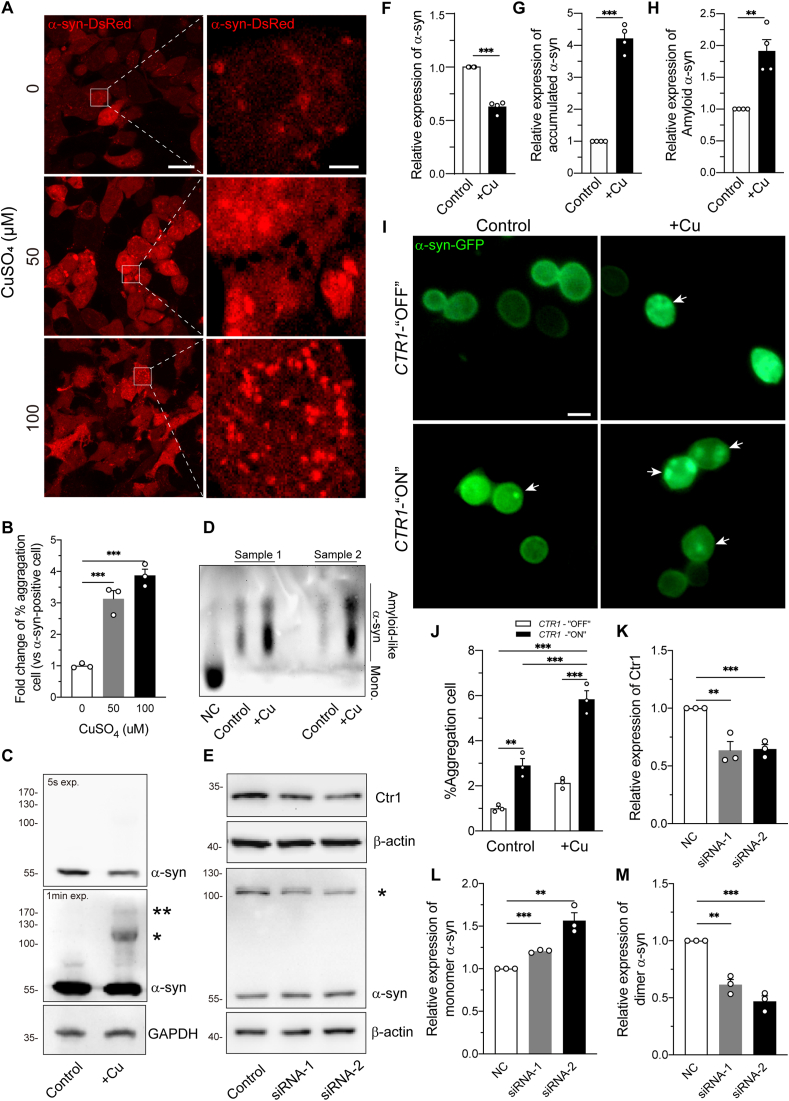
**Ctr1 regulates copper mediated α-syn aggregation in mammalian cells and yeast models**. (A) Confocal microscopy representative pictures showing intracellular α-syn inclusion (left panel, low magnification image scale bar: 200 μm; right panel, high magnification image scale bar: 25 μm). (B) Quantification of the percentage of α-syn accumulated cells (α-syn accumulated cell/α-syn positive cell) is shown in the left panel (n = 500 α-syn positive cells/group). The fold change in accumulated cells increased in Cu group. One-way ANOVA with Tukey's post hoc test. (C) Representative immunoblot of monomer α-syn and accumulated α-syn (marked with stars*^,^ **) in western blot. 20 μg of total protein per line were loaded on 10% SDS-PAGE. The concentration of CuSO_4_ is 50 μM in Cu-group. (D) The amyloid-like protein of α-syn was determined by SDD-AGE. The protein of negative control (NC) group was boiled at 95 °C for 5 min and 200 μg of total protein were loaded per well. The concentration of CuSO_4_ in Cu-groups (Sample 1 and 2) is 50 μM. (E) Representative immunoblots of Ctr1 in siRNA transfection experiment. 20 μg of total protein per line were loaded on 10% SDS-PAGE. (F–H) Quantitative analysis of monomer α-syn (F), accumulated α-syn (G) and α-syn amyloid-like protein (H) relative levels were performed by intensity measurement and normalized to control group. Unpaired *t*-test. (I) Immunofluorescence images of α-syn inclusions (white arrow) in α-syn overexpression yeast strains with 500 μM CuSO_4_ (scale bars: 2 μm). (J) Quantification of % α-syn accumulated cells (α-syn accumulated cell/α-syn positive cell) under different conditions (n = 300 α-syn positive cells/group). Two-way ANOVA with Tukey's post hoc test. (K–M) Quantification of relative expression of Ctr1 (K), monomer α-syn (L) and dimer α-syn (M) was performed by intensity measurement and normalized to control group. Unpaired *t*-test. All data were represented as mean ± SEM of at least three independent experiments. *p < 0.05; **p < 0.01, ***p < 0.001 were considered to be significant.

### 3.2 Ctr1 regulates α-syn pathology in yeast and mammalian models

The high-affinity Cu transporter Ctr1 is crucial for Cu uptake and distribution. We hypothesized that Ctr1 might modulate Cu mediated α-syn aggregation. To address this, we generated the yeast model stably expressing hα-syn fused with GFP, while it was conditionally expressed Ctr1 under control of a galactose-inducible (GAL1) promoter as shown in Supplementary Fig. 2A. Once inducing by galactose, Ctr1 was expressed abundantly in the two yeast strains as shown in Supplementary Fig. 2B. Next, we selected one of the yeast strains (line 2) for the subsequent experiments. The proportion of cells containing α-syn inclusions in the *CTR1*-“ON” group (Ctr1 overexpression) was twice of that in the *CTR1*-“OFF” group (Fig. 1I left panels and Fig. 1J). Furthermore, with high Cu levels, Ctr1 overexpression induced more severe α-syn inclusions (Fig. 1I right panels and Fig. 1J), indicating that Ctr1 overexpression promoted α-syn aggregation. To put it another way, silencing Ctr1 could inhibit α-syn aggregation. To confirm this role of Ctr1 in mammalian cells, we used small interfering RNA (siRNA) to knock down Ctr1 in HEK293 cells stably expressing DsRed-tagged human α-syn. The efficacy of siRNAs targeting Ctr1 was characterized 48h after transfection (Fig. 1E and K). Concomitantly, the level of monomer Ser129-phosphorylation α-syn was increased and the level of oligomer Ser129-phosphorylation α-syn was robustly decreased with siRNA-1 or siRNA-2 transfection (Fig. 1E, L and M), suggesting that silencing Ctr1 inhibited α-syn pathology.

### 3.3 Ctr1 modulates AAV-induced α-syn pathology in the nigrostriatal system

These *in vitro* findings suggest that α-syn pathology can be regulated by Ctr1. Given that Ctr1 decreases in the SNpc in PD patients, we hypothesized that Ctr1 might contribute to α-syn-mediated pathology of PD *in vivo*. In order to verify our hypothesis, we had to generate Ctr1 knockout animal model, but systemic Ctr1 knockout mice were embryonic lethal [44,45]. We thus generated a new mouse model with Ctr1 conditionally knockout (Ctr1-CKO) in the dopaminergic neurons using the Cre-loxP system as shown in Supplementary Fig. 3A–C. Ctr1-CKO mice exhibited no obvious growth or developmental abnormalities. At the same time, we detected that Ctr1 expression decreased (Supplementary Fig. 3D and E) and CCS increased (Supplementary Fig. 3D and G) in the SN of Ctr1-CKO mice. As the CCS level is negatively correlated with Cu concentration in the cytoplasm [35,43], the increase of CCS illustrates Cu reduction in the SN. While the levels of Cu-related proteins (Ctr1, CCS) remained unchanged in the cortex (Supplementary Fig. 3H, I and J). On the other hand, Cu concentrations in brain (cortex, striatum and SN) and liver were indistinguishable from WT mice (Supplementary Fig. 3 K-N). In view of Ctr1 conditionally knockout in the dopaminergic neurons, these results demonstrated the reduction of intracellular Cu in the dopaminergic neurons of Ctr1-CKO mice. Additionally, the level of tyrosine hydroxylase (TH) was unchanged in the SN between genotypes (Supplementary Fig. 3D and F). Likewise, we observed that Ctr1-CKO mice did not exhibit any motor deficits, such as latency to fall in wire hang test and total distance in open field test, and body weight was unchanged at different ages compared to WT mice (Supplementary Fig. 3O, P and Q). Overall, these data indicate that Ctr1 deficiency in the dopaminergic system cannot lead to parkinsonism with motor dysfunction.

To further investigate the role of Ctr1 in α-syn mediated pathology, we used AAV driven overexpression of α-syn in our mice model. The AAV-hα-syn injection can induce α-syn pathologies and progressive loss of dopaminergic neurons and terminals in the nigrostriatal system [61,[66], [67], [68]]. WT and Ctr1-CKO mice were unilaterally injected with AAV-hα-syn or AAV-GFP (as the control) in the SN (Fig. 2A). Eight weeks after viral injections, we examined the expression of α-syn in the nigrostriatal pathway. We found that α-syn-positive signals enriched (approximately 6-fold) in the ipsilateral side of the SN (Fig. 2B, C and D) and striatum (Supplementary Fig. 4A and B) in the AAV-hα-syn injected mice compared to AAV-GFP injected ones. Yet, α-syn immunoreactivity was observed in the contralateral SN and striatum (Fig. 2C, Supplementary Fig. 4A), which might be due to nigrostriatal crossing projections as previously described [[69], [70], [71]]. Furthermore, quantification of α-syn staining showed no difference in the SN or striatum between WT and Ctr1-CKO mice (Fig. 2D, Supplementary Fig. 4B). These data indicate that Ctr1 deficiency cannot affect α-syn expression induced by AAV-hα-syn in the nigrostriatal system.

**Fig. 2:**
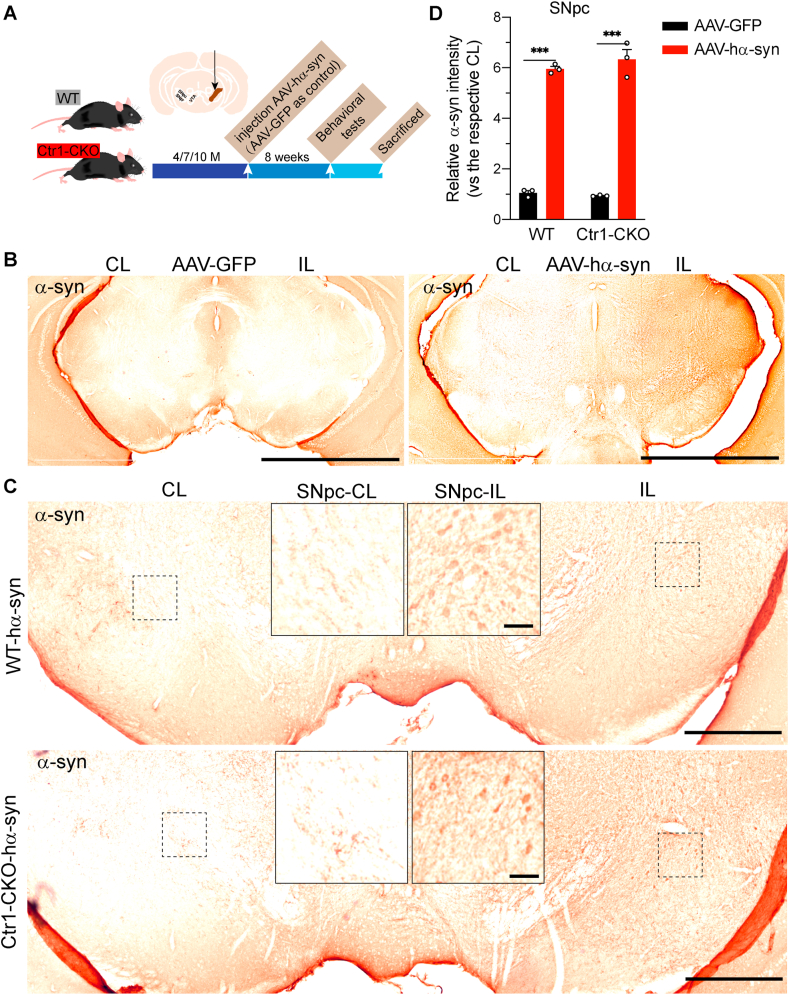
**Ctr1 deficiency cannot affect AAV induced α-syn expression in the SN**. (A) Experimental timeline of unilaterally injected AAV-GFP or AAV-hα-syn into the SN of WT and Ctr1-CKO mice. (B) Illustration of α-syn immunoreactivity in AAV induced expression of α-syn in the SN after injection 8 weeks. AAV-GFP was the control group. Midbrain sections containing the SN were stained by anti-α-syn. The sections were harvested 9 months WT mice after injection of AAV-GFP or AAV-hα-syn. Scale bars: 1000 μm. CL (contralateral), IL (ipsilateral, injected side). (C) Representative images of AAV induced α-syn expression in the SN of 9 months WT and Ctr1-CKO mice after injection AAV-hα-syn. The SNpc-CL (left panel) and SNpc-IL (right panel) images are of higher magnification obtained from the same CL or IL section. Low magnification image (entire image) scale bars: 500 μm; high magnification image (left panel and right panel) scale bars: 50 μm. (D) Quantification of relative α-syn expression in the SN (n = 3 mice/group). Eight images per mouse were used to quantification. Normalized with the AAV-GFP group. Tukey's multiple comparisons test followed two-way ANOVA. Data were represented as mean ± SEM. ***p < 0.001 was considered to be significant.

pS129-α-syn has been suggested as the most common post-translational modification, which was a key pathological feature in PD [14,15]. Double-immunofluorescence staining confirmed that TH-positive neurons of the SN were efficiently infected by AAV-hα-syn (Fig. 3A). To explore the pathological α-syn alterations, we performed immunohistochemistry of pS129-α-syn in both WT and Ctr1-CKO mice with AAV-hα-syn injection. Most of the phosphorylated α-syn positive signals only appeared in the ipsilateral SN of both WT and Ctr1-CKO mice, while the contralateral side had almost no positive signals (Fig. 3A and B). Notably, the number of pS129-α-syn positive cells in Ctr1-CKO mice significantly decreased in the different aged groups compared to the corresponding ones of WT mice (Fig. 3C, decreased by 27% at 6 months, 31% at 9 months, 35% at 12 months). The reduction was age-dependent and most significant at 12months (Fig. 3D). Interestingly, no clear pS129-α-syn positive fibers were observed in the striatum of both WT and Ctr1-CKO mice, which suggested that pS129-α-syn mainly occurred in the soma of neurons rather than in the terminal. In summary, Ctr1 deficiency reduces the deposits of pS129-α-syn in the SN, suggesting that α-syn pathology can be extenuated via downregulation of Ctr1.

**Fig. 3:**
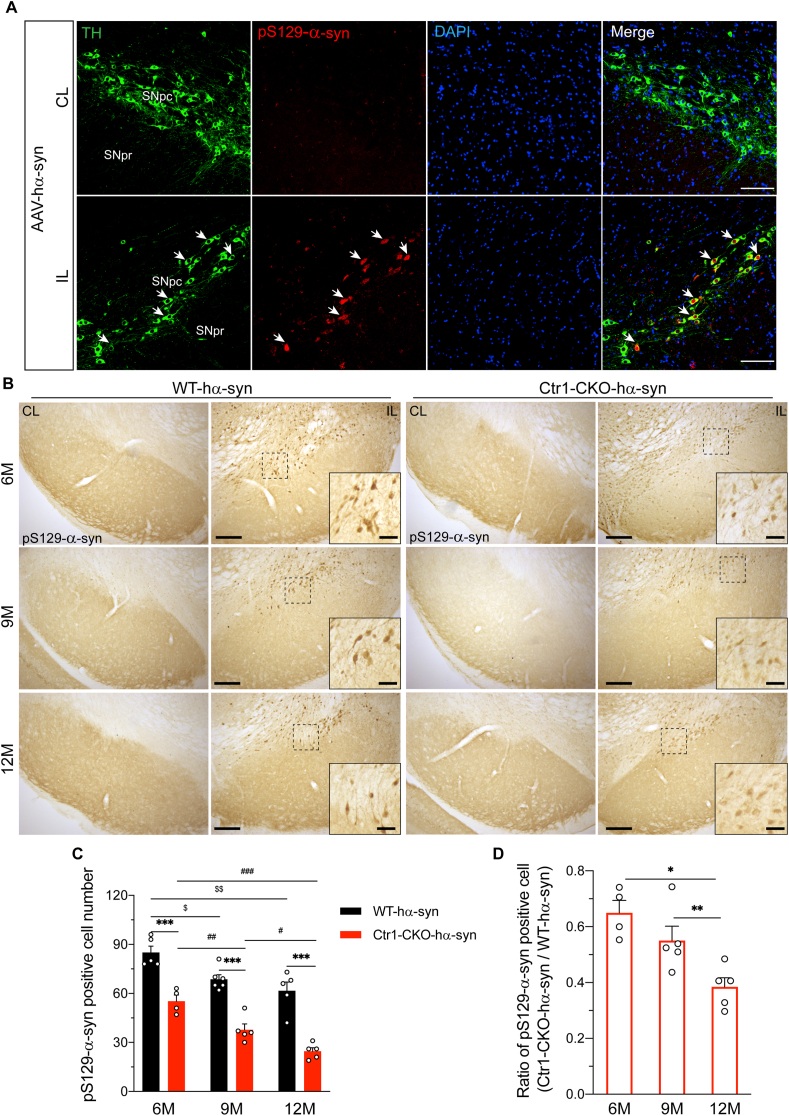
**Ctr1 deficiency reduces the pS129-α-syn positive cells in the SN**. (A) Representative images of double-immunofluorescence staining of pS129-α-syn and TH in the SN of 9 months WT mice injected AAV-hα-syn. The white arrows were shown pS129-α-syn positive cells. CL (contralateral), IL (ipsilateral, injected side), SNpc (substantia nigra pars compacta), SNpr (substantia nigra pars reticulata). Scale bars: 100 μm. (B) Representative images of pS129-α-syn in the SN of 6/9/12 months WT and Ctr1-CKO mice after injection AAV-hα-syn. The signals of pS129-α-syn only appeared in the ipsilateral SN of both WT and Ctr1-CKO mice. The insert images are of higher magnification obtained from the same section. Low magnification image scale bars: 200 μm; high magnification image scale bars: 50 μm. (C) Quantification of pS129-α-syn positive cell number in the SN. The number of pS129-α-syn positive cells in Ctr1-CKO mice significantly decreased (27% at 6 months, 31% at 9 months, 35% at 12 months). WT-hα-syn (n = 5/6/5 mice at 6/9/12 M, respectively); Ctr1-CKO- hα-syn (n = 4/5/5 mice at 6/9/12 M, respectively). Two-way ANOVA with Tukey's post hoc test. (D) The ratio of pS129-α-syn positive cell number (Ctr1-CKO- hα-syn/WT-hα-syn) reduced with age-dependent. One-way ANOVA with Tukey's post hoc test. Data were represented as mean ± SEM. *p < 0.05; **p < 0.01, ***p < 0.001 were considered to be significant.

Recent studies have shown that the deposits of pathological α-syn in LBs or LNs specifically colocalize with cellular protein degradation markers in neurodegenerative disorders, e.g., p62 and ubiquitin, which have failed to efficiently degrade pathological α-syn [11,[72], [73], [74]]. To further study the mechanism that inhibition of Ctr1 downregulates pathological pS129-α-syn in Ctr1-CKO mice, we performed immunofluorescence staining of p62, ubiquitin, and pS129-α-syn in the AAV-hα-syn injected mice. We observed that pS129-α-syn hardly colocalized with p62 in the SNpc of Ctr1-CKO mice but not of WT mice (Fig. 4A). Concordantly, pS129-α-syn also scarcely colocalized with ubiquitin in the SNpc of Ctr1-CKO mice but not of WT mice (Fig. 4B). These data indicate less or no localizations between p62 or Ubiquitin and pS129-α-syn in Ctr1-CKO mice, which suggests that p62 and ubiquitin can successfully target pathological α-syn for cellular degradation, leading to less PD-like symptoms.

**Fig. 4:**
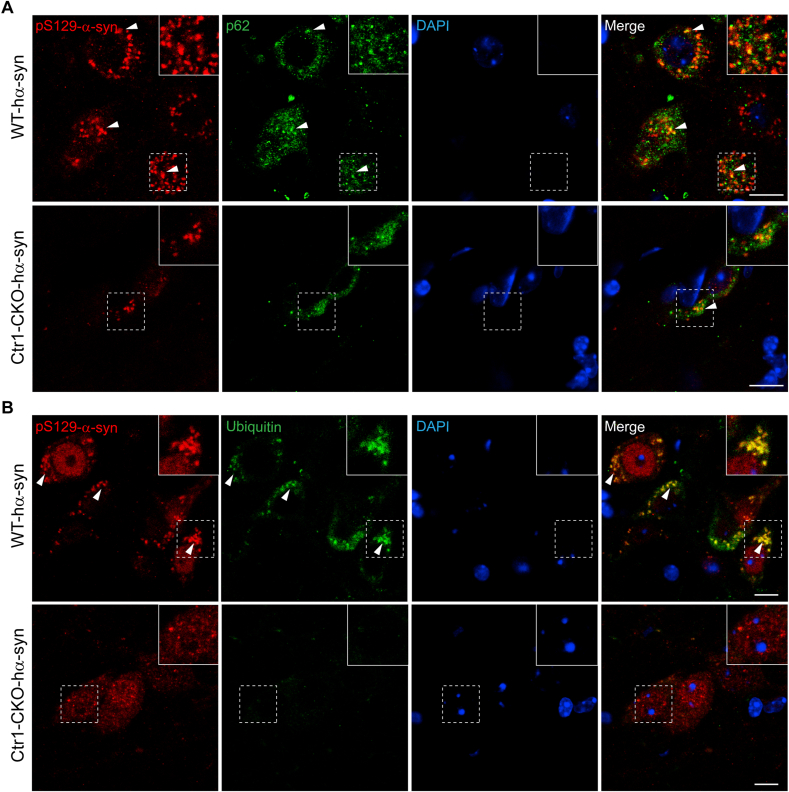
**Deposits of pS129-α-syn colocalize with markers of degradation in the SN**. (A) Representative images of double-immunofluorescence staining of pS129-α-syn and p62 in the SN of 12 months WT and Ctr1-CKO injected AAV-hα-syn mice. The colocalization of p62 and pS129-α-syn (white arrow) decreased in Ctr1-CKO injected AAV-hα-syn mice. (B) Representative images of double-immunofluorescence staining of pS129-α-syn and ubiquitin in the SN of 12 months WT and Ctr1-CKO injected AAV-hα-syn mice. The colocalization of ubiquitin and pS129-α-syn (white arrow) decreased in Ctr1-CKO injected AAV-hα-syn mice. Confocal microscopy images scale bars: 10 μm.

### 3.4 Ctr1 deficiency reverses the TH-positive cells and fibers loss

Studies in animal models of PD have shown that α-syn overexpression induced by viral infection can cause degeneration of the nigrostriatal system [61,67,68]. To see whether Ctr1 deficits can regulate this effect on our models, we evaluated TH-positive signals in the nigrostriatal pathway. Both TH-positive cells in the SNpc (Fig. 5A) and TH-positive fibers in the striatum (Fig. 6A, Supplementary Figs. 5–7) significantly decreased in the AAV-hα-syn injected mice compared to the corresponding ones in the control groups. As we found that the TH positive neurons had a tendency to decrease at old age as previously reported [75], the ratio of TH positive neurons in the injection side to the ones in the contralateral side was performed to show the age-dependent change of nigral neurons. Ctr1 deficits significantly increased the number of dopaminergic neurons to 27% (6 months), 39% (9 months), and 39% (12 months) (Fig. 5B). Consistently, the density of TH-positive fibers also increased in the striatum of Ctr1-CKO mice at different ages (6, 9, 12 months) compared to those of WT mice (Fig. 6B). Collectively, these results agree with the notable reduction of the pS129-α-syn, which indicates that Ctr1 deficiency prevents TH-positive cell and fiber loss from α-syn overexpression-mediated pathology in the nigrostriatal pathway.

**Fig. 5:**
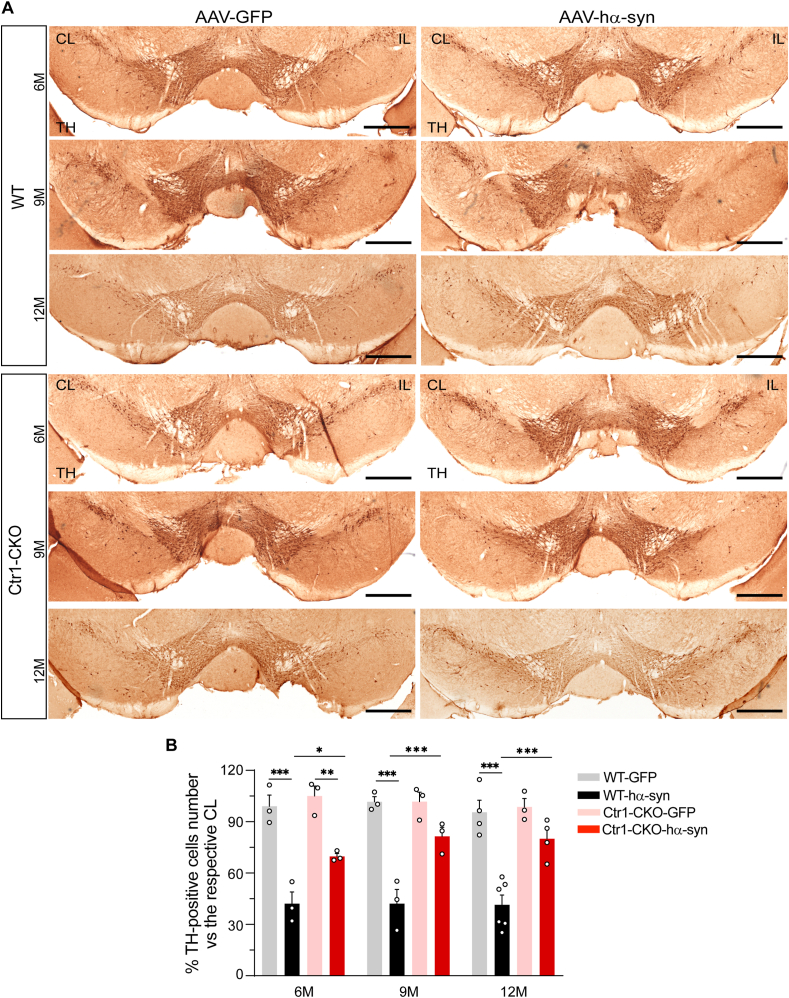
**Ctr1 deficiency rescues the TH-positive cells in the SN**. (A) Representative immunohistochemical images of TH staining in the SN of both WT and Ctr1-CKO mice injected with AAV-hα-syn or AAV-GFP at 6/9/12 months (scale bars: 500 μm). (B) Stereological quantification of the TH-positive cells in the SN. Three sections per mouse were used to stereological counting. Data were shown as a percentage of the respective contralateral side. WT-GFP (n = 3/3/4 mice at 6/9/12 M, respectively); Ctr1-CKO-GFP (n = 3/3/3 mice at 6/9/12 M, respectively), WT-hα-syn (n = 3/3/6 mice at 6/9/12 M, respectively); Ctr1-CKO-hα-syn (n = 3/3/4 mice at 6/9/12 M, respectively). Data were represented as mean ± SEM. Tukey's multiple comparisons test followed two-way ANOVA. *p < 0.05; **p < 0.01, ***p < 0.001 were considered to be significant.

**Fig. 6:**
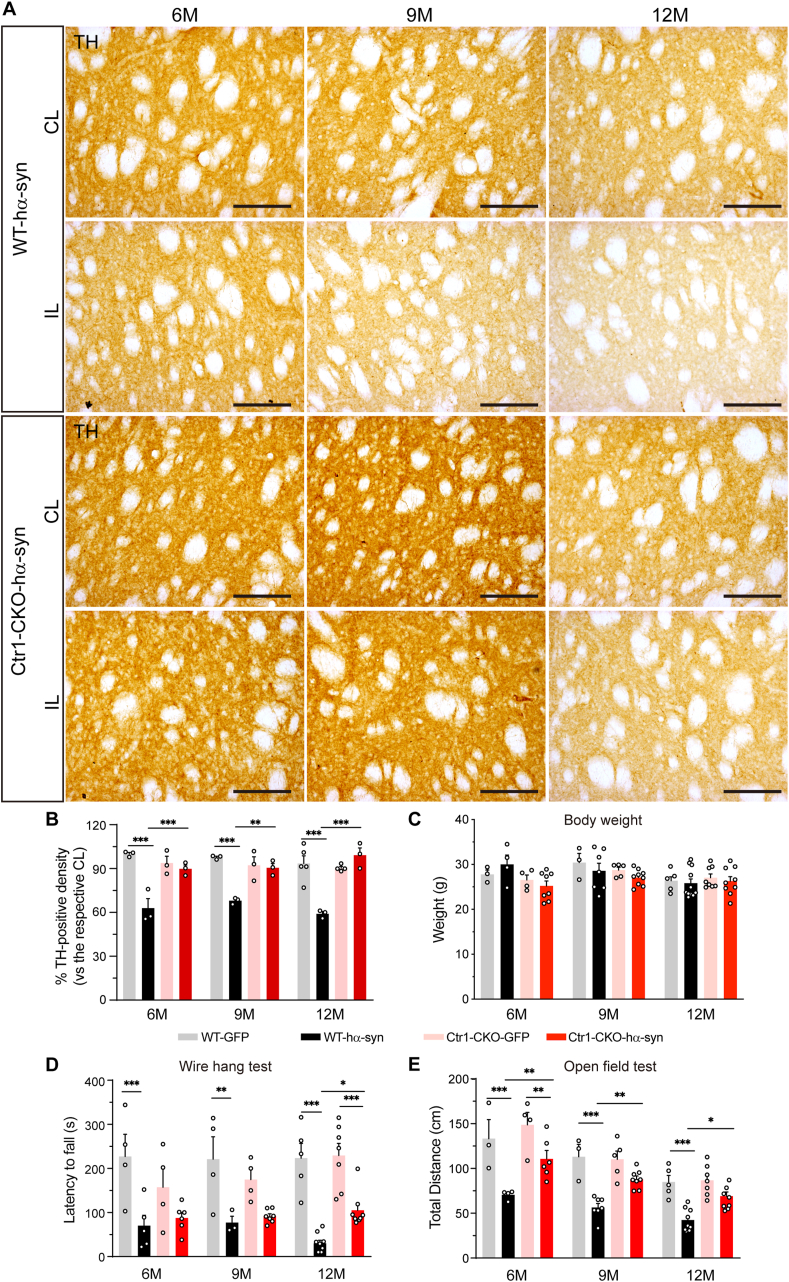
**Ctr1 deficiency rescues the TH-positive fiber loss and alleviates the motor dysfunction**. (A) Images of immunohistochemical staining for TH showing the TH-positive fibers in the striatum at 6/9/12 months in the AAV-hα-syn injected mice (scale bars: 100 μm). The images of AAV-GFP groups were shown in Supplementary Fig. 6. Low magnification images were shown in Supplementary Fig. 5 and Supplementary Fig. 7. (B) Quantification of the TH-positive fibers in the striatum. Eight images per mouse were used to quantification. WT-GFP (n = 3/3/5 mice at 6/9/12 M, respectively); Ctr1-CKO-GFP (n = 3/3/4 mice at 6/9/12 M, respectively), WT-hα-syn (n = 3/3/3 mice at 6/9/12 M, respectively); Ctr1-CKO-hα-syn (n = 3/3/3 mice at 6/9/12 M, respectively). Tukey's multiple comparisons test followed two-way ANOVA. (C) There was no difference in the body weight between WT and Ctr1-CKO mice at 6/9/12 months after injection AAV-GFP or AAV-hα-syn 8 weeks. (D) The latency to fall in wire hang test. At 12 months, the latency to fall increased in Ctr1-CKO-hα-syn mice compared with WT-hα-syn mice. (E) Total distance in the open field test. Compared with WT-hα-syn mice, the total distance increased in Ctr1-CKO-hα-syn mice at 6/9/12 months. Moreover, Ctr1-CKO-hα-syn mice exhibited no significant difference in the open field test compared to Ctr1-CKO-GFP mice. (C–E) It was ensured at least three mice in each group. The number of different groups was provided in Supplementary Table 1. Tukey's multiple comparisons test followed two-way ANOVA. All data were represented as mean ± SEM. *p < 0.05; **p < 0.01, ***p < 0.001 were considered to be significant.

### 3.5 Ctr1 deficiency alleviates motor dysfunctions induced by α-syn overexpression

Progressive neurodegeneration in the nigrostriatal system contributes to motor dysfunction. In the present work, there was no change in mouse body weight at 6, 9, and 12 months of age among all groups (WT-GFP, WT-hα-syn, Ctr1-CKO-GFP, and Ctr1-CKO-hα-syn) (Fig. 6C). At different ages, α-syn overexpression caused motor dysfunctions, i.e., abnormalities of muscle strength in the wire hang test (Fig. 6D) and distinctly decreased spontaneous movement in the open field test (Fig. 6E), irrespective of genotype (both in WT and Ctr1-CKO mice). However, the deficits of muscle strength induced by α-syn overexpression could be rescued in Ctr1-CKO mice at 12 months (Fig. 6D). More importantly, at every different age, the spontaneous movement of Ctr1-CKO mice displayed dramatic increase compared to that of WT mice in the AAV-hα-syn injection groups (Fig. 6E). At 9 and 12 months, Ctr1-CKO mice exhibited no significant difference in spontaneous movement, with or without α-syn overexpression (Fig. 6E). Overall, the behavioral tests showed that Ctr1 deficiency alleviates the motor dysfunction (most significant at 12 months) induced by α-syn overexpression, which is highly consistent with pS129-α-syn decrease (least signal at 12 months) and TH neuron protection (highest survival at 12 months) in Ctr1-CKO mice, indicating that inhibition of Ctr1 prevents the α-syn mediated pathology *in vivo*.

## 4 Discussion

Previous experiments have reported that the level of Ctr1 decreased in surviving neurons in the SN of postmortem PD brains [40], whether this phenomenon is a cause or consequence of PD remains elusive. Our data indicated that the TH level (Supplementary Fig. 3D and F) and TH-positive neurons and fibers (Fig. 5A and B; Fig. 6A and B) were unaffected in Ctr1-CKO mice, implicating that Ctr1 deficiency has no effect on dopaminergic system. Moreover, no significant parkinsonism-related motor dysfunctions were observed in Ctr1-CKO mice, even at advanced ages. Collectively, this study showed that the reduction of Ctr1 is not sufficient to induce PD-like symptoms, which suggests that Ctr1 deficiency may be a consequence or protective reaction of PD pathology. Moreover, our mammalian cell and yeast models showed that silencing Ctr1 inhibits pathological α-syn inclusion. Accordingly, we hypothesize that the reduction of Ctr1 may be involved in a protective mechanism in the pathological development of PD. To test this possibility, we injected AAV-hα-syn to induce α-syn-mediated parkinsonism in Ctr1-CKO mice. The observations that inhibition of Ctr1 ameliorated dopaminergic neuronal neurodegeneration and hampered the motor dysfunction induced by α-syn overexpression favor our hypothesis.

What possible mechanisms underlie Ctr1-related neuroprotection? There are three Cu^2+^ binding sites in α-syn, i.e., a low affinity and specificity C-terminal site (Asp121) and two high specificity N-terminal sites (His50, NH_2_) [26,28]. Cu^2+^ has been reported to accelerate α-syn aggregation by these binding sites *in vitro* [23,27,30]. Besides, α-syn has other three anchoring sites with Cu^+^ including the lowest affinity sites Met116 and Met117, the higher affinity site His50 and the highest affinity sites Met1 and Met5 [27,29,[63], [64], [65]]. These specific sites of Cu binding α-syn have been proposed to play a role in α-syn aggregation [29]. It is worth noting that the physiological α-syn is easily N-terminally acetylated *in vivo* [76], which caps the Cu^2+^ higher-affinity binding sites [77]. Therefore, N-acetylated α-syn mainly interacts with Cu^+^ through two methionine residues at the N-terminal (Met1 and Met5) [63,64]. Meanwhile, Cu^+^ is the predominant state of copper ions under physiological conditions in living cells [27,64]. The Cu^+^ complex with N-acetylated α-syn increases and stabilizes local conformations with helical secondary structure and restricted motility [64,78]. As Ctr1 transports Cu across the plasma membrane with high affinity and specificity, the depletion of Ctr1 efficiently reduces the intracellular Cu level [43,79,80], mainly the Cu^+^ level [79]. According to our *vitro* and *vivo* results, low level of intracellular Cu thus forms fewer Cu-α-syn complexes producing fewer α-syn aggregates. α-Syn aggregates are considered the pathological hallmark of PD and cause a series of neurodegeneration and neurotoxicity-related processes, such as mitochondrial impairment, oxidative stress, synaptic dysfunction, defective endoplasmic reticulum (ER) function and autophagy pathway, and nuclear dysfunction, as previously reviewed [12]; fewer α-syn aggregates result in less α-syn mediated toxicity and pathology [81]. Thus, less α-syn aggregation improves neuron viability. Additionally, the Cu-α-syn complex alters the redox properties of Cu and enhances oxidative stress (e.g., H_2_O_2_, GSH) leading to cell death [82]. Depletion of Ctr1 leads to reduction of both the Cu and Cu-α-syn complexes, possibly decreasing oxidative stress induced by the Cu-α-syn complex to prevent dopaminergic neuronal death. However, the associated pathways and molecular mechanisms remain poorly understood and deserve further investigation.

Different approaches have been attempted to explore potentially therapeutic strategies for PD and other related synucleinopathies, e.g., modulating the α-syn level [83,84] and interfering with α-syn aggregation or/and degradation [[85], [86], [87]]. Regarding Cu-mediated α-syn aggregation, application of Cu chelators partially reduced the nitration and fibrillation of α-syn in animal models of PD [88,89]. Due to extracellular treatment, chelators reduce the Cu level both in neurons and non-neuronal cells (e.g., astrocytes). Cu deficiency in astrocytes can cause reduction of ceruloplasmin, which is mainly charged with iron export [90,91]. This subsequently induces iron deposition in astrocytes, which eventually leads to a series of cell dysfunction and neurotoxicity-related processes in the brain [48,92]. Cu chelators thus are not ideal to treat Cu-mediated α-syn aggregation. In contrast, modulating the Cu levels through Cu transporters, such as Ctr1, ATP7A, ATB7B, etc, becomes better options [35,47]. The importation of Cu into cells is mainly dependent on Ctr1. ATP7A and ATP7B are involved in Cu secretion. As we know, Cu is an essential metal element for the activity of cells. Systemic Ctr1 depletion is embryonic lethal and intestinal epithelial cell-specific Ctr1 knockout exhibit peripheral Cu absorption defects in mouse experiments [43,45,79]. Thus, Ctr1 is crucial to function and healthy of cell as the Cu transporter. According to our data, regulation through Ctr1 may be more specific and harmless, as Ctr1 is an endogenous Cu regulatory protein and manipulating its expression can efficiently control the intracellular Cu level [43,80,93]. Furthermore, the application of particular promoters (e.g., *αCaMKII, DAT*) can ensure genomic modification in a unique type of cell [[94], [95], [96]]. Our observations (i.e., no change in the Cu level in the SN and cortex, the dramatic rise of CCS, and the significant reduction of Ctr1 in the SN) (Supplementary Fig. 3) indicate that Cu decreases in dopaminergic neurons, whereas the integral Cu metabolism remains unchanged in the SN, suggesting that Cu metabolism is regulated very precisely and with spatiotemporal specificity in the brain. Overall, genetically handing Ctr1 has become a potential method to modulate Cu-mediated α-syn pathology.

Notably, the X-ray crystal structure of Ctr1 has revealed a homo-trimeric Cu-selective ion channel-like architecture with two methionine triads forming a Cu selectivity filter near the extracellular entrance [42,97]. This enables designing or selecting small molecule compounds targeting the extracellular Cu binding sites to modulate the intracellular Cu level. Further, advanced techniques provide more alternative methods to efficiently silence Ctr1, such as siRNA (the present work), short hairpin RNA (shRNA) [80], and CRISPR-Cas9 mediated gene editing [98]. Additionally, new technologies, such as synchrotron-based x-ray fluorescence microscopy [40] and new fluorescent copper sensors for confocal or two-photon imaging [99], can detect Cu concentration in real time at the single-cell level, which combined with new gene editing of Ctr1 may help develop precise treatments. Although the detailed mechanisms underlying the neuroprotective function of Ctr1 remain to be further elucidated. These cutting-edge techniques, together with our theoretical work, provide immense possibilities to ameliorate α-syn-mediated parkinsonism through targeting Ctr1.

To conclude, our findings highlight Ctr1 as a potential molecular target to intervene Cu-mediated α-syn aggregation and pathology and extend a broader view for therapy of Cu dysmetabolism-related neurodegenerative diseases, such as AD, ALS, PD, and other synucleinopathies.

## Consent for publication

All authors have read the manuscript and indicated consent for publication.

## Data availability statement

All data generated or analyzed during this study are included in this published article (and its supplementary information files).

## Funding

Financial supports by the 10.13039/501100001809National Natural Science Foundation of China (81430025, 31800898, and U1801681 to J.Y.L. and W.L., 31870140 to C.D.) and the Key Field Research Development Program of Guangdong Province (2018B030337001); and The 10.13039/501100004359Swedish Research Council (K2015-61X-22297-03-4; 2019–01551), EU-10.13039/100013278JPND research (aSynProtec and REfrAME) and EU-Horizon2020 (MSCA-ITN-2016, SynDeGen), ParkinsonFonden, the Strategic Research Area Multipark (Multidisciplinary research in Parkinson's disease at 10.13039/501100003252Lund University), the Fundamental Research Funds for Central Universities of China (N172002001 to C.D.), and the Xing Liao Program (XLYC1807001 to C.D.)

## Author contributions

D.H.G., T.T.H., C.D., and J.Y.L. designed research; D.H.G., T.T.H., X.D.G., X.H.W., D.Y.S., and C.H. performed research. D.H.G., X.L., L.Z., and Y.T. performed the stereological counting. D.H.G., and T.T.H. analyzed and interpreted the data; D.H.G., T.T.H., W.L., C.D., and J.Y.L. wrote the manuscript. All authors edited and approved the paper.

## Ethics approval

Ethical approval was granted from the Research Ethics Committee of 10.13039/501100004184Northeastern University, Shenyang, China. All involving animal experiments and procedures were approved by the Ethical Committee for the use of laboratory animals at Northeastern University, China.

## Declaration of competing interest

All the authors state that they do not have any conflict of interests.

## Figures and Tables

**figs1:**
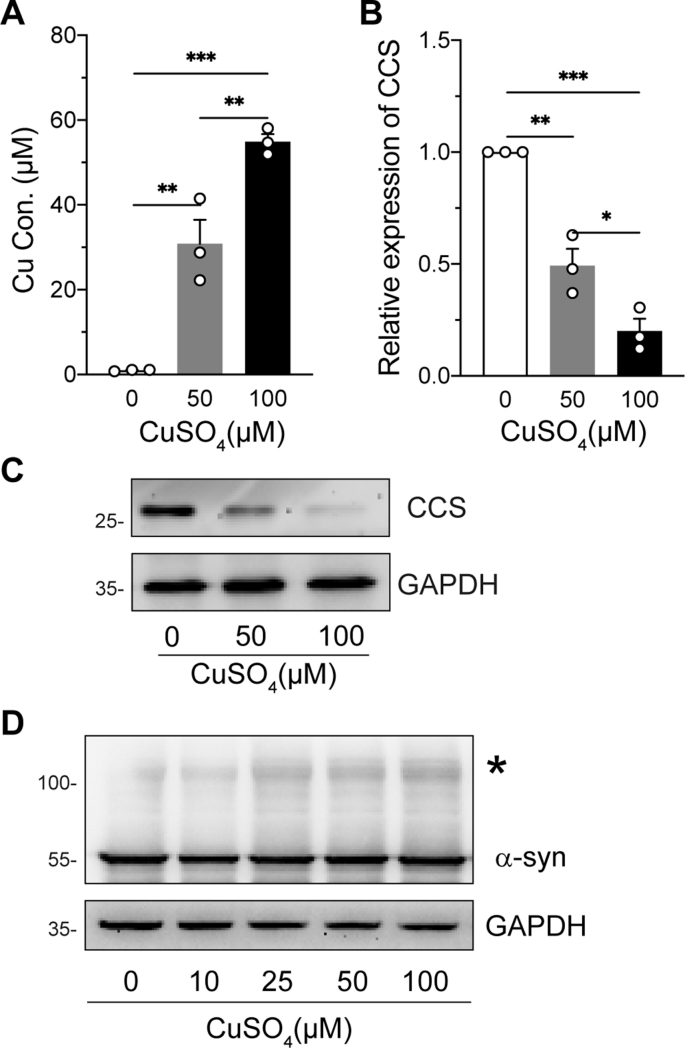


**figs2:**
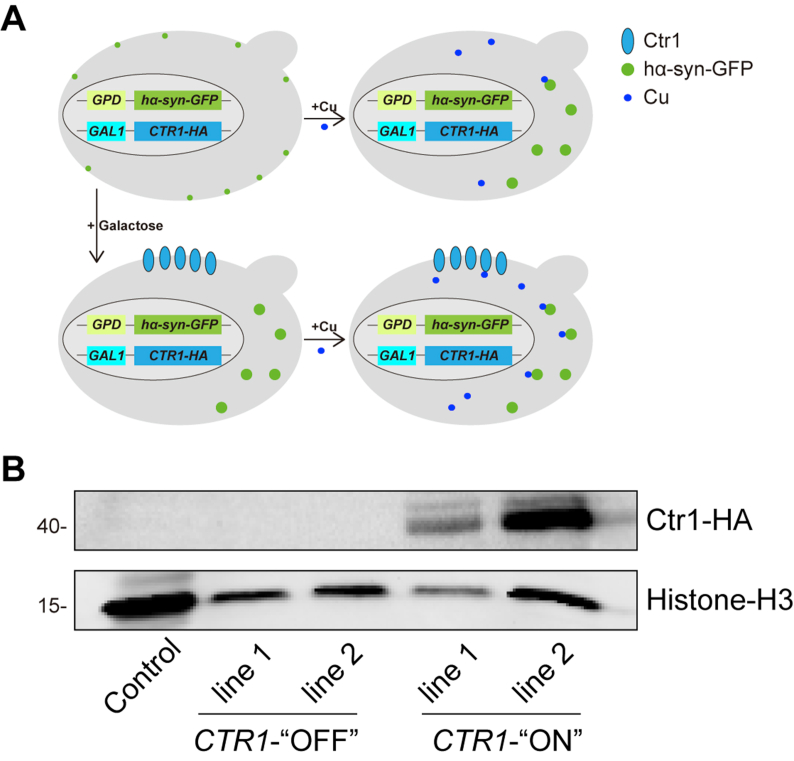


**figs3:**
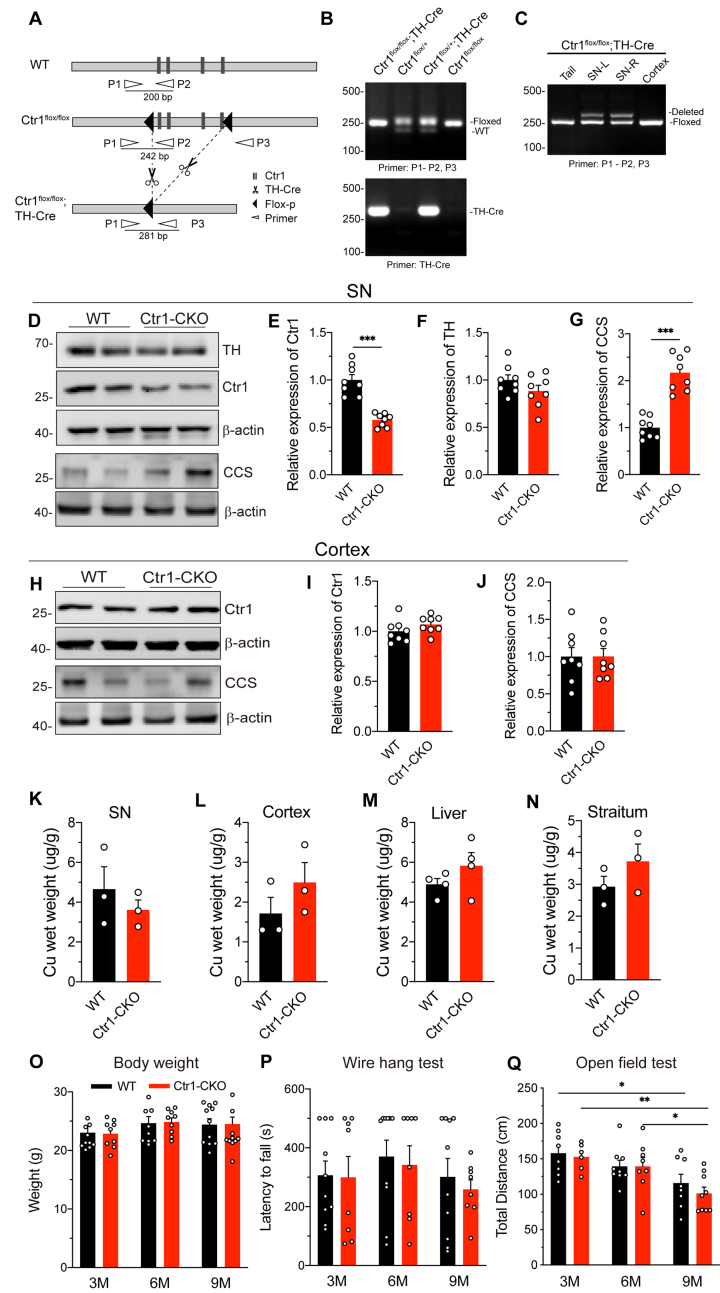


**figs4:**
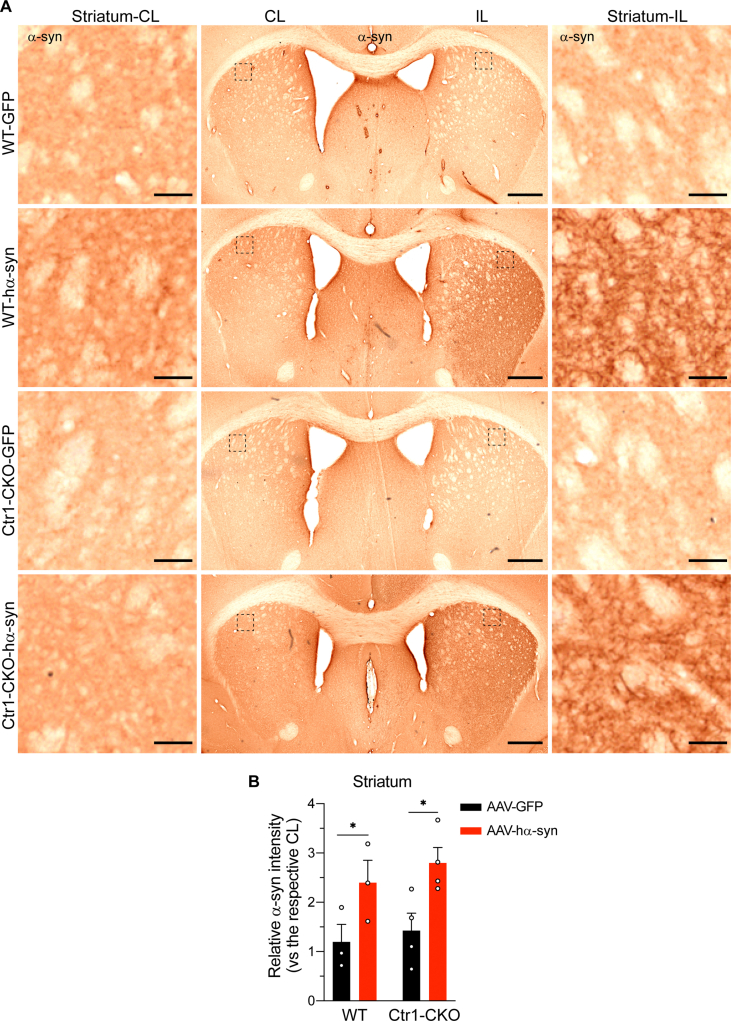


**figs5:**
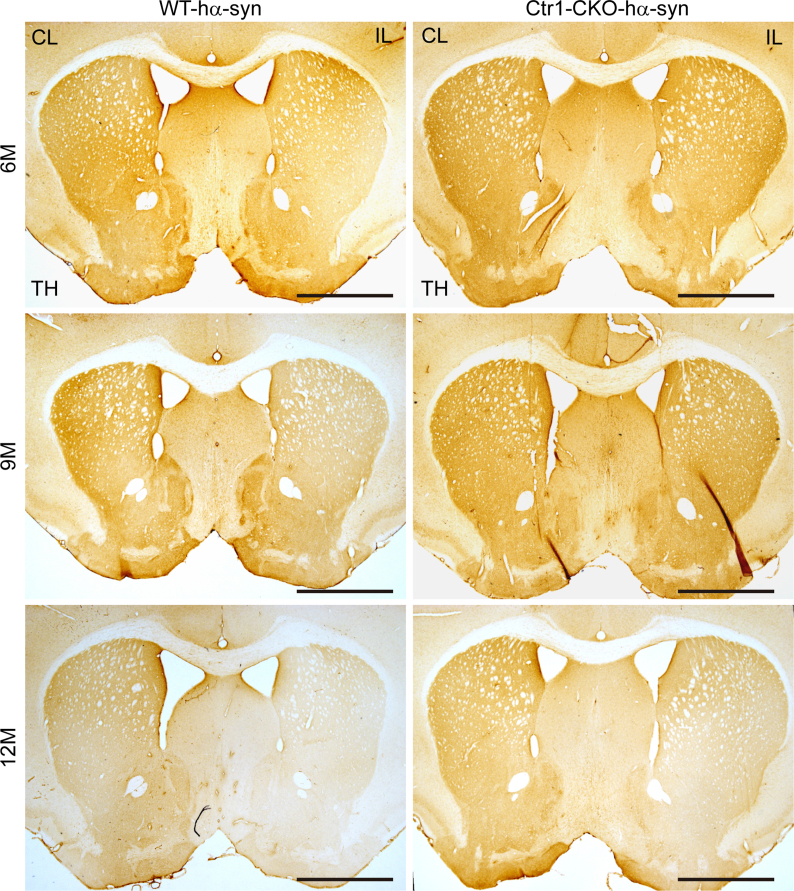


**figs6:**
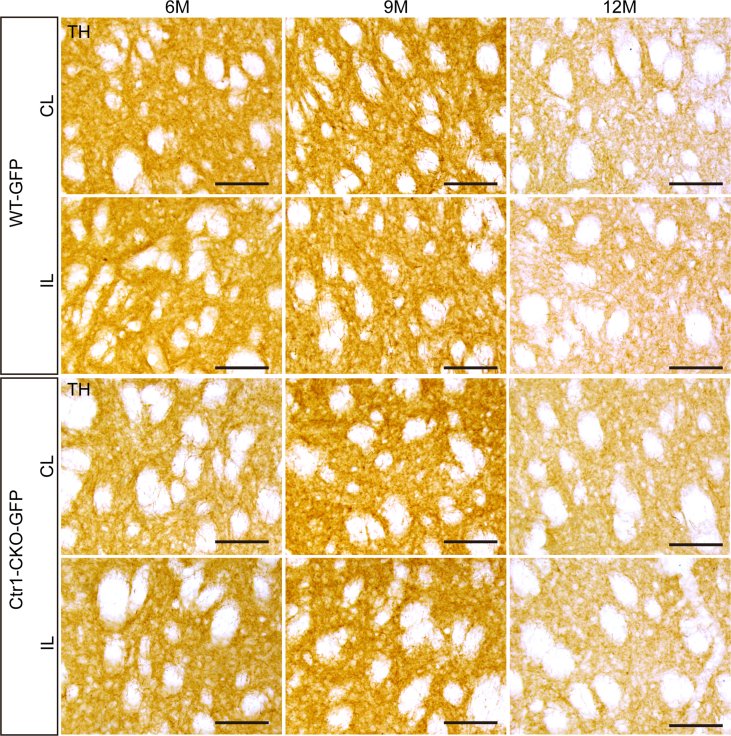


**figs7:**